# Monitoring of Structures and Mechanical Systems Using Virtual Visual Sensors for Video Analysis: Fundamental Concept and Proof of Feasibility

**DOI:** 10.3390/s131216551

**Published:** 2013-12-02

**Authors:** Thomas Schumacher, Ali Shariati

**Affiliations:** Civil and Environmental Engineering, University of Delaware, Newark, DE 19716, USA; E-Mail: alish@udel.edu

**Keywords:** structural health monitoring, video analysis, natural vibrations, virtual visual sensors, Eulerian specification

## Abstract

Structural health monitoring (SHM) has become a viable tool to provide owners of structures and mechanical systems with quantitative and objective data for maintenance and repair. Traditionally, discrete contact sensors such as strain gages or accelerometers have been used for SHM. However, distributed remote sensors could be advantageous since they don't require cabling and can cover an area rather than a limited number of discrete points. Along this line we propose a novel monitoring methodology based on video analysis. By employing commercially available digital cameras combined with efficient signal processing methods we can measure and compute the fundamental frequency of vibration of structural systems. The basic concept is that small changes in the intensity value of a monitored pixel with fixed coordinates caused by the vibration of structures can be captured by employing techniques such as the Fast Fourier Transform (FFT). In this paper we introduce the basic concept and mathematical theory of this proposed so-called virtual visual sensor (VVS), we present a set of initial laboratory experiments to demonstrate the accuracy of this approach, and provide a practical in-service monitoring example of an in-service bridge. Finally, we discuss further work to improve the current methodology.

## 1. Introduction

Video-based methods have recently been introduced for a variety of applications in structural health monitoring (SHM). Patsias and Staszewski [1] analyzed digital videos for edge detection and to approximate the mode shape of a cantilever in a laboratory experiment. By applying a wavelet transform to the mode shape they were able to detect the location of damage which was introduced by cutting a groove with increasing depth into the cross-section. Lee *et al.* [2] devised a real-time method to measure in-plane displacements and rotations using feature tracking techniques based on a Lagrangian approach, and applied it to a target bridge. Zaurin and Catbas [3–7] developed a method using digital video data to locate and measure applied loads on a bridge and devised an index called unit influence line (UIL) as a measure of the health of bridges. Elgamal *et al.* [8] developed a framework to integrate different data types including computer vision data to create a “decision-support system” for bridges and other lifelines. In a SHM review on wind turbines by Ciang *et al.* [9], it is noted that digital image correlation (DIC) techniques can also be used for these structures, but the 3-D version of these methods should be investigated in more depth if they are to be applied. Song *et al.* [10] modified the Hough Transform to track numerous markers on a beam with a computationally efficient algorithm and fitted a spline curve to the tracked shape in order to detect the location of the damage.

To conclude, the use of digital videos for SHM is only in the beginning stage. With the availability of inexpensive yet high-quality digital video cameras we believe there is great potential that has not been fully explored yet. The methodology we present in this paper uses commercially available camera technology combined with an efficient and simple methodology to capture and compute structural vibration data from digital videos.

## 2. Motivation

The objective of this study was to evaluate a novel sensing approach for structural health monitoring (SHM) purposes which is contactless, inexpensive, and flexible in its application. Vibration data are important in a number of disciplines such as mechanical and structural engineering. A comprehensive review on structural health monitoring (SHM) shows the efforts put forth to estimate damage and damage location based on observed changes in natural frequencies of vibration [11]. The literature contains different resources addressing vibration-based SHM as well [12–18]. Finally, natural frequencies from in-service structures are often used to calibrate finite element (FE) models [19,20].

## 3. Proposed Sensing Approach

### 3.1. Background

In a recent inspiring paper on Eulerian video magnification, Wu *et al.* [21] present an innovative yet beautifully simple approach to magnify subtle motions in digital videos so that they become visible to the naked eye. This was done using an Eulerian specification where a pixel with a fixed coordinate is selected and its value monitored in time. In contrast, in a Lagrangian specification one would attempt to track a specific feature in a video in time and space. One of the examples presented, which may have great potential for application in the medical field, measures the pulse of a person by analyzing a video taken from the person. The inventors found that the minute change in intensity in the red content, *R*, of the person's skin was significant enough to be analyzed to accurately compute the person's pulse. Another example was a video of a person's wrist where the expansion and contraction of the veins were amplified to be clearly visible. The advantage is that this approach is contactless and can be performed continuously without interfering with the person. Motivated by this article we introduce here a methodology based on the same fundamental idea for potential use in the field of structural health monitoring (SHM) for structures and mechanical systems.

### 3.2. Methodology

We propose that every pixel in a digital video taken from a structure represents a candidate virtual visual sensor (VVS) that may be used for SHM purposes (first suggested by Patsias and Staszewskiy [1]). The term “VVS” follows the terminology suggested by Song, Bowen *et al.* [10]. Although the approach presented in the latter paper may appear similar, it is fundamentally different as they were employing a Lagrangian specification where a target (or feature) is tracked in space and time.

**Figure 1.**
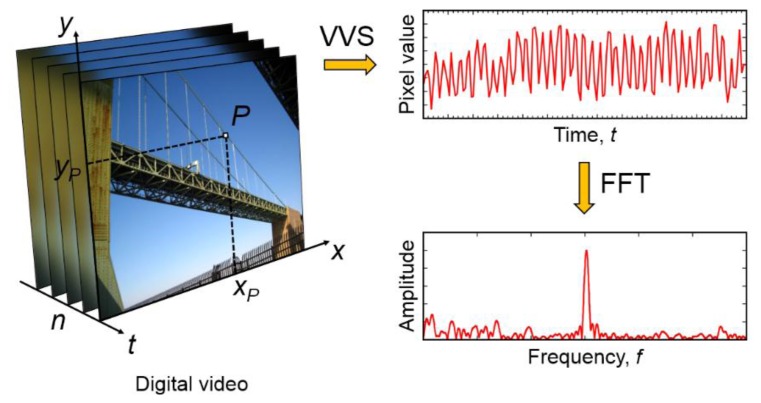
Proposed methodology of a virtual visual sensor (VVS) to measure structural vibrations. *x_p_* and *y_p_* represent fixed coordinates of the monitored pixel, *P*.

Our proposed methodology uses an Eulerian specification where a specific pixel is selected and monitored which is illustrated in Figure 1; the intensity of the pixel at location *x_p_* and *y_p_* is monitored over time and analyzed using the Fast Fourier Transform (FFT) [22,23] to reveal the fundamental frequency of vibration. Note that the pixel value in the time domain represents gray-scale intensity and does not directly correspond to the amplitude of vibration, *i.e.*, displacement. As a result, at this point we are not able to estimate the amplitude but only the frequency of the motion which represents a limitation.

### 3.3. Theoretical Basis

Digital videos are a sequence of digital images captured at a specified frame rate. Typical frame rates of commercially available cameras are 25, 30, or 60 fps (=frames per second). In this study we used a range of cameras, including two inexpensive point-shoot cameras with 25 and 30 fps, respectively, and a new high-speed camera mainly used in the adventure sports community that can capture videos up to 240 fps. Video frames are typically stored in *RGB* (red-green-blue) color mode as measured by the camera's image sensor [24]. A single grey-scale value, called intensity, *I*, is assigned to each pixel where 0 and 255 represent black and white, respectively. MATLAB uses a linear combination to calculate *I* based on *RGB* values that eliminates hue and saturation information while retaining the luminance [25,26]:

(1) I=0.2989R+0.5870G+0.1140B

**Figure 2.**
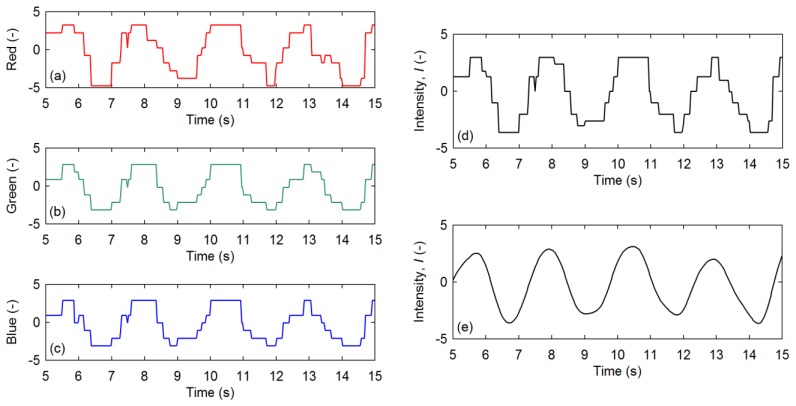
Example of experimental data extracted from a VVS: brightness of (**a**) red; (**b**) green; and (**c**) blue; (**d**) computed intensity (used for subsequent analyses); I, and (**e**) smoothed intensity (for illustrative purposes).

An example of experimental data extracted from a VVS and the resulting intensity curves are shown in Figure 2. If the intensity value is smoothed using a 5-point moving average as shown in Figure 2e, the quantization effects that exist in the raw brightness values (Figure 2c) and the computed grey-scale intensity curve (Figure 2d) can effectively be removed to reveal a relatively harmonic motion. For this study, only raw intensities (example shown in Figure 2d) were used for the computation of frequencies.

**Figure 3.**
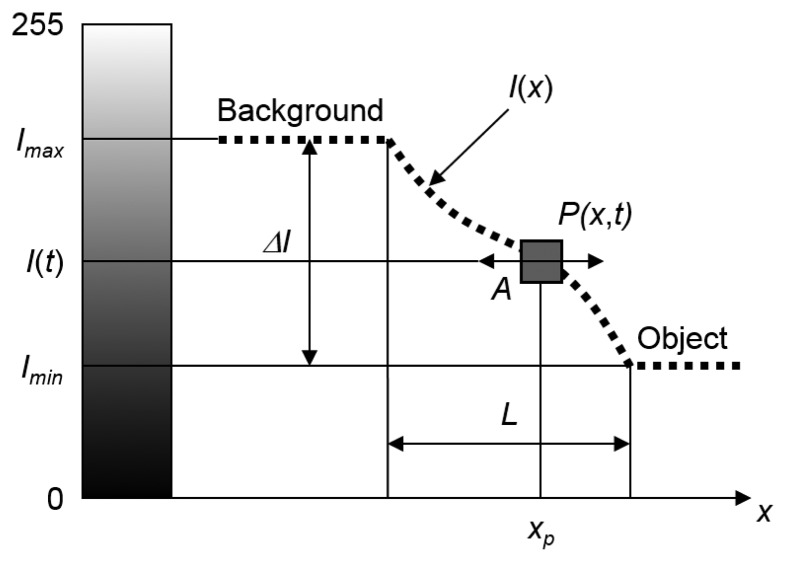
Illustration of the relationship between vibration of motion in direction *x* (one direction only for simplification), amplitude of vibration *A*, pixel location *x_P_* and pixel size, and shape of the grey-scale intensity curve *I*(*x*).

Figure 3 illustrates the factors that influence the accuracy and reliability of the proposed VVS. The dotted line represents the grey-scale intensity curve *I*(*x*) along a path *x*. For this theoretical example, the background is assumed to be light colored and the object of interest dark colored. The location and size of the monitored pixel is depicted by the grey square denoted with *P*(*x*,*t*). *L* represents the length over which the intensity changes. Figure 3 represents a snapshot and as time *t* progresses the intensity curve *I*(*x*) will vibrate horizontally (in the x-direction) with an amplitude *A* causing the pixel to oscillate vertically about *x_p_*, following the *I*(*x*) curve.

If the object, characterized by the intensity *I*(*x*), is vibrating at a natural frequency *ώ*_0_ and without losing generality we can write *x*=*A*sin(*ω*_0_*t*), hence the intensity value becomes *I*(*x*)=*I*(*A*sin(*ω*_0_*t*)). If we want to consider the effect of the function *I*(*x*) on our measured peak frequencies we can write:

(2) $$F\left(I\left(x\right)\right)=\int_{-\infty }^{\infty }I\left(A\sin\left(\omega_{0}t\right)\right)e^{-j\omega t}dt$$

If *I*(*x*) is a linear function and by subtracting the DC term we are able to compute the exact peak frequency (neglecting any quantization noise) *I*(*A*sin(*ω*_0_*t*))=*C* sin(*ω*_0_*t*) so that:

(3) $$F\left(I\left(t\right)\right)=\frac{\pi }{j}C\left[\delta \left(\omega -\omega_{0}\right)+\delta \left(\omega +\omega_{0}\right)\right]$$

where *C* is a constant. If *I*(*x*) is a nonlinear function, e.g., *x^n^*, with the identity $\sin\left(\omega_{0}t\right)=\frac{e^{j\omega t}-e^{-j\omega t}}{2}$ we can write:

(4) $$F\left(I\left(t\right)\right)=\int_{-\infty }^{\infty }{\left(A\frac{e^{j\omega t}-e^{-j\omega t}}{2}\right)}^{n}e^{-j\omega t}dt={\left(\frac{A}{2}\right)}^{n}\int_{-\infty }^{\infty }\sum_{k=0}^{n}{\left(-1\right)}^{k}\left(\begin{array}{c} n\\ k \end{array}\right)e^{-j\left(n-2k\right)\omega_{0}t}e^{-j\omega t}dt$$

Taking the summation Σ out of the integral we obtain:

(5) $$F\left(I\left(t\right)\right)=2\pi {\left(\frac{A}{2}\right)}^{n}\sum_{k=0}^{n}{\left(-1\right)}^{k}\left(\begin{array}{c} n\\ k \end{array}\right)\delta \left(\omega -\left(n-2k\right)\omega_{0}\right)$$

Equation (5) reveals that any nonlinearity of degree *n* in *I*(*x*) produces peak frequencies at (*n* – 2*k*)*ώ*_0_ for 0 < *k* < *n*. It is important to note that if *I*(*x*) can be written as a power series, the magnitude of the spurious impulses in the frequency domain can be calculated based on the above equation. The extreme positions (peak amplitude points) of the VVS with respect to the intensity curve *I*(*x*) should be located on an approximately linear portion of *I*(*x*) and within *L*. If *I*(*x*) is nonlinear, spurious frequency peaks will occur as can be observed in Figure 5b which will be explained in more detail in Section 4.1. From the discussion above, the following can be concluded:

- The intensity range Δ*I*=(*I*_max_−*I*_min_) should be maximized, *i.e.*, a small range will increase quantization noise. This can be achieved by selecting proper background and lighting conditions.
- The number of pixels across *A* should be maximized which is directly related to the spatial resolution.
- The amplitudes of vibration should be small, *i.e.*, the maximum amplitude of vibration *A* should lie within *L* in order to avoid the appearance of nonlinear system behavior.
- The size of the pixel (or VVS) with respect to the length should be small to avoid averaging of measured intensity values and additional quantization noise.

Additionally, the following factors influence the accuracy of the VVS:

- High sampling rates, *i.e.*, a large number of frames per second, decrease the quantization noise. Minimum sampling rates as given by the Nyquist-Shannon sampling theorem [27] apply and are discussed in Chapter 4.
- The total signal duration *T* directly influences the resolution and thus the uncertainty of the VVS, *i.e.*, the resolution of a signal in the frequency domain is Δ*f* = *T*^−1^.
- Finally, moving and shaking of the camera, changing illumination, and noise in the image sensor influence the signal-to-noise ratio and therefore the accuracy of the computed peak frequency.

## 4. Experimental Verification

**Figure 4.**
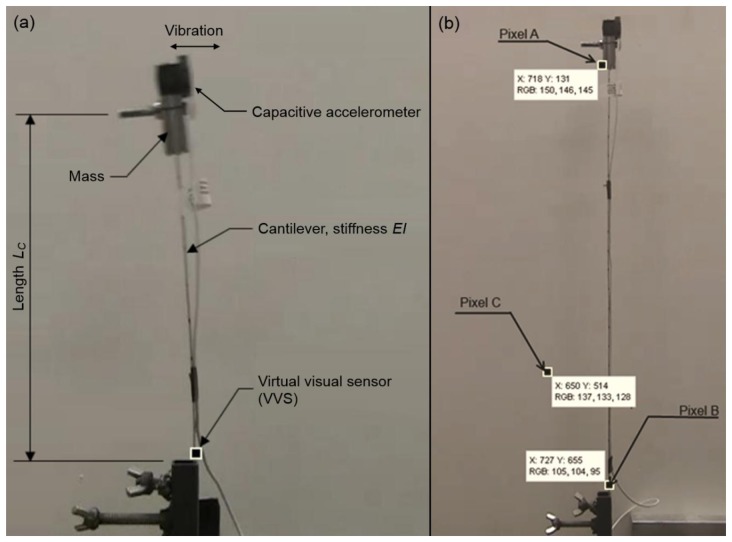
(**a**) Snapshot of moving cantilever and (**b**) candidate virtual visual sensors (VVS).

To verify the validity and accuracy of our proposed approach, a cantilever beam with adjustable length, *L_C_* (*i.e.*, variable stiffness) and a concentrated constant mass on top, equivalent to a single-degree-of-freedom (SDOF) system [28], was tested as shown in Figure 4a. The test was initiated by creating an initial displacement (by hand) and then letting the cantilever vibrate in its natural mode of vibration. Acceleration was measured using a high-accuracy capacitive accelerometer (Model 2260-010 by Silicon Designs, Inc., Kirkland, WA, USA, sampling at 1 kHz) attached to the mass. Additionally, a digital video was taken during the test capturing the motion of the cantilever using two different cameras: a commercially available digital camcorder (Model ViXIA HFS100 HD by Canon USA, Inc., Melville, NY, USA, recording at 30 fps) for frequencies up to 10 Hz and a relatively high-speed camera (Model Hero 3 by Woodman Labs, Inc., Half Moon Bay, CA, USA, recording at 120 fps) for higher frequencies. It should be noted that, as for any digitally sampled signal, the Nyquist-Shannon [27] sampling theorem applies, *i.e.*, the sampling rate needs to be set to at least twice the highest anticipated frequency to be distinguishable in the signal [29]. Anti-aliasing filters were set to one half of the selected sampling frequency for the accelerometer. For the cameras, such an option is currently not available, and one of our goals was to determine whether this represents a problem. The selection of the pixel to be monitored (candidate VVS) turned out to be critical to obtain meaningful frequency data from the videos and a thorough discussion is included in the following section.

### 4.1. Candidate Virtual Visual Sensors

The advantage that every pixel in the video represents a candidate virtual visual sensor (VVS) is also the challenge. In this section we compare and discuss the signals computed from a number of different candidate pixels. For this evaluation the cantilever length, *L_C_* was kept constant at 25 in (635 mm). Figure 4b shows three candidate VVS: Pixels *A* and *B* are located near the top and the bottom of the cantilever where the largest and smallest amplitudes of vibration occur, respectively. Pixel *C* is located away from the cantilever but capturing its shadow. Intuitively one might pick pixel *A* since it is located where the largest motion takes place which should produce the best data. However, in the case of our proposed approach this does not work well as it is discussed next.

**Figure 5.**
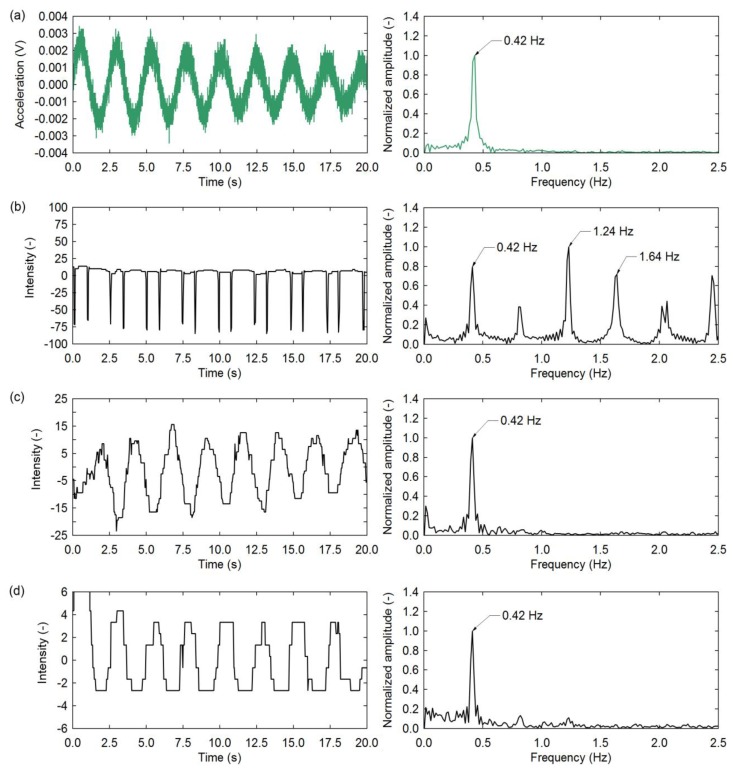
Time history (left column) and frequency (right column) data for (**a**) accelerometer; (**b**) pixel *A*; (**c**) pixel *B*; and (**d**) pixel *C*. Note: the intensity time histories were centered about 0 vertically to avoid a large DC component in the frequency domain.

Figure 5a shows example data collected with the accelerometer. Although pixel *B* is at the bottom of the cantilever, a place with the smallest motion which can hardly be observed by the naked eye, the change of intensity (grey scale pixel value) is represented by a relatively harmonic signal (Figure 5c). For pixel *A* located near the top of the cantilever, where the amplitude of vibration is largest, the intensity value experiences periodic impulses due to the sudden occlusion of the mostly grey background by the beam. As a result, the FFT produces a periodic function as well, showing pronounced harmonic peaks, as can be observed in Figure 5b. This can be mathematically explained (see Section 3.3) by a highly non-linear function such as *I*(*x*) = *x^n^* where *n* is a large number. Although the peak frequency is present and correct, the upper harmonics are very strong as well which makes the analysis more difficult. Alternatively, pixel *C* is found to produce a relatively harmonic signal as well (Figure 5d). Although it is not located on the structure, it can capture the motion of its shadow. This represents an opportunity to observe vibrations indirectly, in case the actual structure is not directly observable. These facts suggest that a reliable candidate VVS for measuring frequency is a point with small amplitudes of vibration near the physical boundary (*i.e.*, edge) of the structure where the change in intensity is most pronounced (a theoretical discussion is presented in Section 3.3). For the cantilever study this can also be a point near the top of the cantilever after damping has reduced the amplitudes of vibration significantly. For practical purposes, this condition is usually satisfied considering the camera is relatively far from the structure compared to the amplitude of vibrations to be captured [13].

Although the total average amplitude of vibration for the data shown in Figure 5a,c was approximately 100 mm and 0.3 mm, respectively, the signal-to-noise ratios for the frequency plots are comparable. This further highlights the potential of this sensing approach to capture small vibrations.

### 4.2. Accuracy of Virtual Visual Sensors

**Figure 6.**
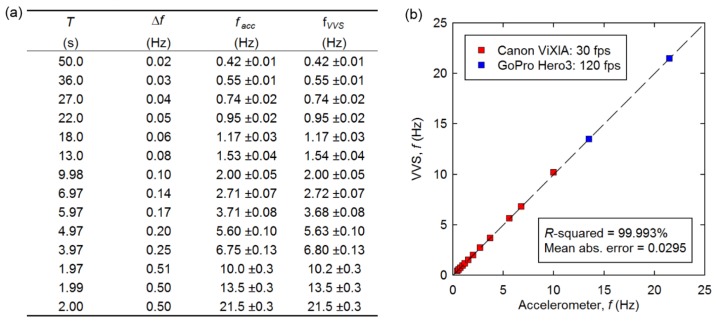
(**a**) Table and (**b**) plot showing correlation between physical accelerometer and virtual visual sensor (VVS).

In order to determine the accuracy of our proposed approach, a pixel close to the bottom of the cantilever was selected to compute the frequency as described previously and shown in Figure 4a. The length, *L_C_* was varied between 50 and 635 mm to produce a range of natural frequencies. Figure 6 shows the correlation between the physical accelerometer and the frequencies computed from the selected VVS. The computed frequencies listed in Figure 6a are given as *f* ± Δ*f*/2 to account for the uncertainty where Δ*f* = 1/*T* with *T* being the duration of the original signal length in seconds. Although we used zero padding to run the FFT in some cases, which will provide smoother peaks in the low frequency range, higher accuracy is not achieved. As can be observed in Figure 6b, there is excellent correlation between the frequencies computed from the two measurements. The squared correlation coefficient and standard error between accelerometer and the camera's computed frequency were found to be 99.993% and 0.0295, respectively.

### 4.3. Frequency Analysis over Range of Pixels

**Figure 7.**
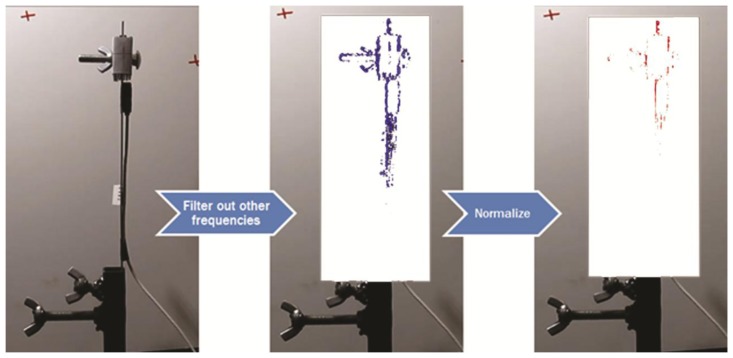
(**a**) Snapshot of cantilever; (**b**) snapshot with highlighted pixels of same peak frequency; and (**c**) snapshot with normalized highlighted pixels of same peak frequency.

An extended approach to determine candidate VVS is to analyze a selected area of pixels in a video around the vibrating structure and then highlighting the pixels that have the same frequency in the image as illustrated in Figure 7. It should be noted that this only works well for small amplitudes of vibration for reasons discussed in Section 4.1. This involves the following steps:

(1) Select range of pixels to be analyzed within video (shown as white box in Figure 7b,c).
(2) Compute time history of intensity values for each of the selected pixels.
(3) Compute the peak frequency for each pixel as described in Section 3.2 and create a histogram.
(4) Highlight the pixels with the same peak frequency in the selected range (Figure 7b).
(5) Normalize the color values with the magnitude of the FFT transform to reduce noise (optional, Figure 7c).
(6) Repeat steps 1 to 5 if more than one significant frequency is present in the histogram.

As can be observed from Figure 7, the result of this analysis is essentially an image of the outline of the vibrating parts of the structure. Note that this was done for a period where the cantilever was experiencing small amplitudes of vibration to avoid problems as discussed in the previous section. This analysis could also be used to average peak frequencies from several measurements rather than using one measurement.

## 5. In-Service Monitoring Example

In order to examine the applicability of this method for practical purposes, a video of an existing major bridge in Oregon was evaluated. The bridge consists of a continuous steel truss and some of the vertical hangers have experienced extensive torsional vibrations due to the high transverse winds which caused vortex shedding. Concerns regarding fatigue at the connections have been raised and as a result, the State Department of Transportation has recently retrofitted some of the susceptible members. The reason for the vibrations is the low torsional stiffness of the used I-sections. In a recent research project the problem was investigated in the laboratory to make predictions on the remaining fatigue life [30].

**Figure 8.**
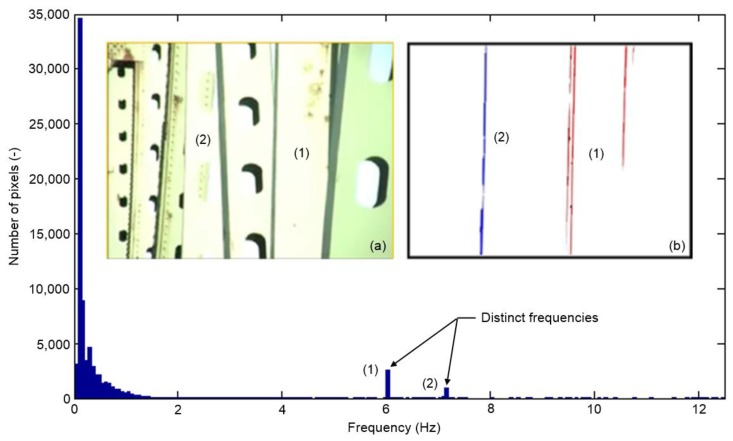
Histogram of peak frequencies from all pixels in the bridge video. Insert: (**a**) Snapshot of video clip; (**b**) Colored pixels with same frequencies.

A video taken by DOT personnel showing torsional oscillations was made available to us. The movie was taken with an inexpensive point-and-shoot-type camera recording at 25 fps by hand (no mechanical stabilization) with the intent to qualitatively document such an occurrence and not necessarily for quantitative analysis purposes. The video captured severe torsional vibrations of two vertical hangers labeled (1) and (2) in Figure 8a, simultaneously. It should be noted that only four seconds from the original video were usable which directly influences the resolution in the frequency domain. For this analysis, all pixels were analyzed and their peak frequency values computed as described in the Section 4.3. Figure 8 shows a histogram of all computed peak frequencies. As can be observed, the majority of computed peak frequencies are close to zero which essentially means that the majority of the pixel intensity values don't change. Frequencies between 0 and 1 can be associated with the fact that the camera was held by hand and therefore probably not completely fixed. A closer look reveals that there are two distinct frequency peaks that can be associated with the torsional vibrations of the two hangers.

By filtering out pixels that are not within the desired frequency range, we obtain the outline of the oscillating hangers (Figure 8b) as described in Section 4.3. The frequencies computed from one selected pixel for members (1) and (2) are 6.1 ± 0.125 and 7.1 ± 0.125 Hz, respectively. A finite element (FE) analysis of a hanger modeled after one of these two members [30] predicted a torsional vibration frequency of approximately 6.5 Hz. This result is not the actual measurement but proves that our computed frequencies obtained from the VVS are plausible. This demonstrates that, although the movie was never taken with the intent for analysis with our proposed methodology, we were still able to deduce useful frequency of vibration information.

## 6. Conclusions and Outlook

The concept of virtual visual sensors (VVS) offers new opportunities for structural health monitoring of structural and mechanical systems. The following conclusions can be drawn from this study:

- The fundamental frequency of vibration of single-degree-of-freedom (SDOF) systems [28] can be accurately computed using the proposed methodology of virtual visual sensors (VVS).
- VVS are inexpensive non-contact sensors with great application flexibility.
- Multiple independently vibrating elements in one video can be distinguished and their fundamental frequency of vibration computed.
- The accuracy and resolution of the measurements depends on a variety of factors such as sampling rate, quantization noise (function of pixel size and location with respect to intensity curve), image sensor quality and size, and lens type.
- By highlighting the pixels with a distinct frequency, the outline of the vibrating elements in a video can be recovered.

In the future we plan to apply the methodology to multi-degree-of-freedom (MDOF) and continuous systems [28]. Furthermore, we want to investigate approaches that not only give frequency but also amplitude of vibration. Eventually, our goal is to establish sensitivity and reliability measures for a range of structural and mechanical applications.
